# Correlation between coronary artery disease severity, left ventricular mass index and carotid intima media thickness, assessed by radio-frequency

**DOI:** 10.1186/1476-7120-9-32

**Published:** 2011-11-16

**Authors:** Marco M Ciccone, Pietro Scicchitano, Annapaola Zito, Luciano Agati, Michele Gesualdo, Sandro Mandolesi, Rosa Carbonara, Francesco Ciciarello, Francesco Fedele

**Affiliations:** 1 Cardiovascular Diseases Section, Department of Emergency and Organ Transplantation (DETO), University of Bari, Bari, Italy; 2 Department of Cardiovascular, Respiratory and Morphologic Sciences of "Umberto I" Polyclinic of Rome, "Sapienza" University, Rome, Italy

**Keywords:** ^RF^QIMT, LVMI, coronary stenosis, CAD, cardiovascular risk

## Abstract

**Background:**

Intima-media thickness of the common carotid artery (CCA-IMT) is a validated marker of systemic atherosclerosis process. The aim of this study was to evaluate the association between coronary artery disease (CAD), left ventricular hypertrophy (LVH) and CCA-IMT, assessed by Radio Frequency-Quality Intima Media Thickness (^RF^QIMT) method, the next generation of IMT real-time measurement, based on the direct analysis of the radiofrequency signal and endowed with high accuracy and reproducibility in early detection of arterial wall thickness.

**Methods:**

115 patients (76 men, mean age: 65.1 ± 12 years) referred to our department and shown significant (≥ 70% luminal obstruction) stenosis at least in one major epicardial coronary artery were studied. Coronary angiograms were divided for severity and extent of the disease: 79 patients (69%) had one, 24 patients (21%) two, 12 patients (10%) three major epicardial coronary arteries with ≥ 70% stenosis. All patients underwent echocardiography and carotid ultrasound examination, assessed by RF.

**Results:**

Dividing ^RF^QIMT data in tertiles, dyslipidaemia (31 patients with IMT ≥ 1.20 mm vs 16 with IMT = 0.91-1.19 vs 25 with IMT ≤ 0.9, p = 0.004), LVMI (153.5 ± 20.6 g/m^2 ^in IMT ≥ 1.20 mm vs 131.2 ± 8.4 g/m^2 ^in IMT = 0.91-1.19 mm vs 114.3 ± 11.1 g/m^2 ^in IMT ≤ 0.9 mm, P < 0.001) and number of high stenosed coronary arteries (IMT ≥ 1.20 mm population more often showed three vessel diseases than IMT ≤ 0.90 mm one, P < 0.001) seemed to be significantly related to CCA-IMT increases. Furthermore, LVMI is positively related to IMT (r = 0.91; P < 0.001). In a multivariate regression model (R^2 ^= 0.88), ^RF^QIMT remained significantly associated with the dyslipidemia (regression coefficient ± standard error [SE]: 0.057 ± 0.023; p = 0.017), LVMI (regression coefficient ± SE: 0.01 ± 0.001; P < 0.0001) and number of damaged coronaries (regression coefficient ± SE: 0.0174 ± 0.028; P < 0.0001).

**Conclusions:**

^RF^QIMT is a sophisticated method for carotid ultrasound evaluation. Its evaluation in patients with at least one important major epicardial coronary vessel stenosis would help the accuracy in the general assessment of the number of coronary lesions in these patients.

## Background

Atherosclerosis and its complications are the worldwide major causes of death. Its pathogenesis deals with inflammation and autoimmune aspects and is well developed in literature [1]. It begins its negative evolution since teenage period of life. This consideration leads physicians to realize several tools and techniques for early detection of the disease [2]. The measurement of common carotid artery intima media thickness (CCA-IMT) is well-established cardiovascular risk marker. Apart from the clear and pragmatic "Mannheim Carotid Intima-Media Thickness Consensus (2004-2006)" [3], many other works put on evidence the real importance of such a vascular ultrasound parameter both in the early detection of first alteration of peripheral vessels [4,5] and in the prediction of the risk of coronary artery disease [6-8]. Ultrasound CCA-IMT evaluation is a general measure of the severity of atherosclerosis, and increased IMT is related to generalized atherosclerosis, although the today state-of-heart method is the evaluation of it by Radio Frequency-Quality Intima Media Thickness (^RF^QIMT) method. Many data [9-11] outlined the comparison between older ultrasound and novel radiofrequency assessing methods, but poor data are in literature about clinical application of the new method. Literature data [12,13] demonstrated the association between carotid IMT and coronary atherosclerosis status, although the assessment had been performed by ultrasound and not by radio-frequency (RF). Moreover, an association between carotid atherosclerosis and cardiac remodelling has also been reported in hypertensive patients without coronary artery disease [14]. Left ventricular hypertrophy (LVH) is a powerful independent risk marker for cardiovascular complications in essential hypertension. A limited number of studies have examined the prevalence, the association and the correlation between modifications and remodelling in the heart and in the vasculature [15], and, furthermore, no one adopted RF to assess vascular markers. At this regard, the novelty of our study was to investigate the association between coronary artery disease (CAD), LVH and CCA-IMT, using ^RF^QIMT method to evaluate carotid arteries.

## Materials and methods

### Study population

We studied 262 patients referred to the Department of Cardiovascular, Respiratory and Morphologic Sciences of "Umberto I" Polyclinic of Rome, "Sapienza" University, between May 2007 and May 2009 for coronary angiography. We recruited patients affected by Acute Coronary Syndrome (unstable angina, non-ST segment elevation myocardial infarction [NSTEMI], ST segment elevation myocardial infarction [STEMI]). We excluded patients in critical conditions who needed urgent revascularization therapy and could not be evaluated by echocardiography and ^RF^QIMT before invasive evaluation and/or treatment. The decision to perform coronary angiography was in accordance with published guidelines [16-19], but we considered eligible for our study only the patients who could undergo to coronary angiography within 72 hours from admission (not considering includable patients who underwent coronary angiography after such a period from the admittance). A second selection had been considered after coronary angiography: we included in our study only the patients that showed significant (≥ 70% luminal obstruction) stenosis at least in one major epicardial coronary artery (Left Anterior Descending Artery, Left Circumflex Artery, Right Coronary Artery) during invasive evaluation. For all these reasons only 115 (76 men), aged 35-85 years (mean age 65.1 ± 12 years) were definitely recruited. Table 1 and 2 collect the clinical status details of the patients definitely enrolled in the study.

**Table 1:** Prevalence of cardiovascular risk factors in the study population.

	Total population	Male Gender	Female Gender
**All patients**	115	76 (66.1%)	39 (33.9%)
**Age (years)**	65.1 ± 12	64.8 ± 12	65.6 ± 12
**Hypertension**	86 (74.8%)	58 (50.4%)	28 (24.4%)
**Diabetes mellitus**	31 (27.0%)	17 (14.8%)	14 (12.2%)
**Dyslipidaemia**	72 (62.6%)	48 (41.7%)	24 (20.9%)
**Light smokers**	11 (9.6%)	5 (4.4%)	6 (5.2%)
**Medium smokers**	45 (39.1%)	29 (25.3%)	16 (13.8%)
**Heavy smokers**	59 (51.3%)	46 (40.2%)	13 (11.0%)
**CAD family history**	30 (26.1%)	21 (18.3%)	9 (7.8%)
**Echocardiography**			
- **LVMI (g/m^2^)**	133.5 ± 21.6	137.1 ± 21.8	126.5 ± 19.7
- **E.F. (%)**	50.0 ± 8.5	50.3 ± 8.7	49.5 ± 8.2
- **IVS (mm)**	12.0 ± 0.9	12.0 ± 0.9	12.0 ± 0.8
- **PW (mm)**	12.2 ± 0.8	12.2 ± 0.9	12.2 ± 0.8
- **LVEDD (mm)**	54.5 ± 3.3	54.4 ± 3.3	54.5 ± 3.4
- **LVESD (mm)**	38.2 ± 4.8	38.0 ± 4.8	38.7 ± 4.8

Mean values ± Standard Deviation or number (percentages) of patients

CAD: coronary artery diseases; LVMI: left ventricular mass index; EF: Ejection Fraction; IVS: interventricular septum; PW: Posterior Wall; LVEDD: Left ventricle end diastolic diameter; LVESD: Left ventricle end sistolic diameter

**Table 2:** Demographic and clinical characteristics of patients according to IMT groups.

	All	IMT < 0.90	IMT 0.91-1.19	IMT > 1.20	
	**n = 115**	**n = 37**	**n = 38**	**n = 40**	**p**

**Age (years)**	65 ± 12	63 ± 15	65 ± 12	67 ± 9	0.33
**Male gender**	76 (66)	20 (54)	28 (74)	28 (70)	0.16
**Smoking**	89 (77)	28 (76)	32 (84)	29 (73)	0.45
**CAD family history**	86 (75)	27 (73)	25 (66)	34 (85)	0.14
**Diabetes mellitus**	31 (27)	10 (27)	8 (21)	13 (33)	0.52
**Dyslipidaemia**	72 (63)	25 (68)	16 (42)*	31 (78)#	**0.004**
**Hypertension**	86 (75)	26 (70)	30 (79)	30 (75)	0.69
**Unstable angina**	30 (26)	14 (38)	10 (26)	6 (15)	0.07
**NSTEMI/STEMI**	85 (74)	23 (62)	28 (74)	34 (85)	0.07
**LVMI (**g/m^2^)	133.5 ± 21.6	114.3 ± 11.1	131.2 ± 8.4	153.5 ± 20.6	**< 0.001**
**Coronary angiography**					
RCA	69 (60)	16 (43)	20 (53)	33 (83)	**0.001**
LAD	26 (23)	7 (19)	5 (13)	14 (35)	0.06
LCX	41 (36)	10 (27)	10 (26)	21 (53)*#	**0.022**
PDA	25 (22)	4 (11)	8 (21)	13 (33)	0.07
LMCA	2 (2)	0 (0)	1 (3)	1 (3)	0.62
**Significantly (> 70%) stenosed coronaries**					**< 0.001**
**Monovasal**	79 (69)	37 (100)	32 (84)*	10 (25)*#	
**Bi-vasal**	24 (21)	0 (0)	6 (16)	18 (45)	
**Tri-vasal**	12 (10)	0 (0)	0 (0)	12 (30)	
**PCI**	32 (28)	6 (16)	6 (16)	20 (50)*#	**< 0.0001**
**CABG**	6 (5)	0 (0)	1 (3)	5 (13)*#	**0.033**

Mean values ± Standard Deviation or number (percentages) of patients.

*P < 0.05 vs. IMT ≤ 0.90; #P < 0.05 vs. IMT 0.91-1.19.

IMT: Intima-media thickness; CAD: coronary artery diseases; RCA: Right coronary artery; LAD: Left Anterior Descending Artery; LCX: left circumflex artery; PDA: posterior descending artery; LMCA: left main coronary artery; PCI: Percutaneous coronary intervention; CABG: Coronary artery bypass surgery; LVMI: left ventricular mass index; NSTEMI: Non-ST segment elevation myocardial infarction; STEMI: ST segment elevation myocardial infarction

The written consent was obtained from each patient in order to be included into the study. This latest one was approved by the Ethics Committee of the "Sapienza" University of Rome and carried out in accordance with the principles of the Helsinki Declaration.

Before coronary angiography, all patients underwent routine examinations: complete clinical history and echocardiography evaluation. Before coronary angiography, all patients underwent two-dimensional echo-color-Doppler of the carotid arteries, adopting a high definition vascular echograph: Esaote MyLab 50 X-Vision, in order to detect with radio-frequency the IMT of carotid artery. Plaque was defined as a focal structure encroaching into the arterial lumen of at least 0.5 mm or 50% of the surrounding IMT value, or demonstrated a thickness greater than 1.5 mm as measured from the media-adventitia interface to the intima-lumen interface [3].

### Patients clinical evaluation

Clinical characteristics of the study patients are given in Table 1. We recorded risk factors profile of each patients: hypertension, diabetes, dyslipidaemia, previous coronary arteries diseases history. In particular, arterial hypertension was diagnosed on the basis of pre-existing treatment with antihypertensive drugs or the criteria published in 2007 European Society of Cardiology guidelines on Arterial Hypertension [20]: i.e. systolic (SBP) and diastolic blood pressure (DBP) levels of ≥ 140 and ≥ 90 mmHg. Diabetes was defined on the basis of serum fasting glucose levels > 126 mg/dl or insulin or oral anti-diabetic drugs treatments. In addition, in accordance with the NCEP-ATP III [21] hypercholesterolemia was defined as total cholesterol ≥ 220 mg/dL or the use of lipid-lowering drug(s). However, all patients included in the study were in pharmacological treatment (anti-hypertensive drugs, statins, oral hypoglycaemic agents) for their own cardiovascular risk factors. We documented also the smoking history of each patient. The patient was considered to be a current daily smoker if he had regularly smoked at least 5 cigarettes/day during the previous 3 months or had stopped smoking less than 1 year before his/her admittance to the our department. We classified as *light smokers *smoking < 10 packs-years, as *medium smokers *those smoking 10-20 packs-years, and as *heavy smokers *those smoking > 20 packs-years.

### Carotid Ultrasonography Assessment

^RF^QIMT method is a well validated [10,11,22,23] method based on the direct analysis of the radiofrequency signal directed towards vessels walls and it could be considered as gold standard technique for diameter, changes in diameter and wall vessel measurements according with its high spatial resolution. All the examinations were performed by the same physician in order to reduce bias.

**Figure 1:**
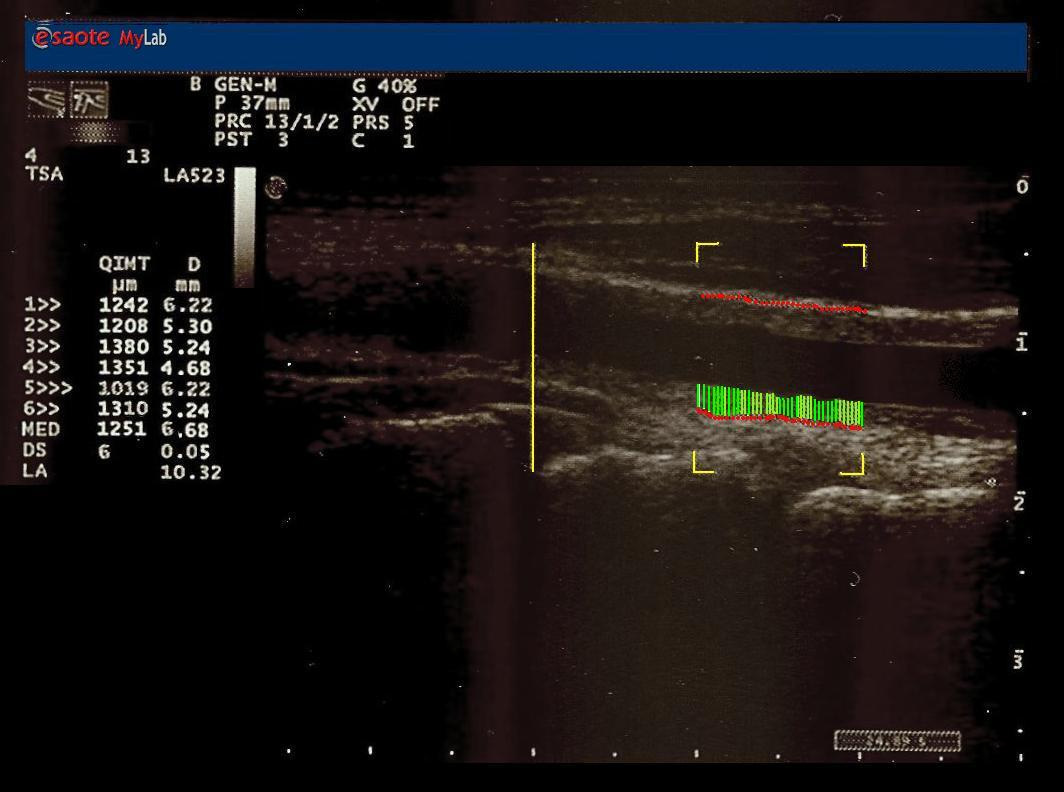
**A sample case of ^RF^QIMT evaluation**.

The normal arterial wall is composed by two acoustic impedance interfaces: the transition between blood and intima, and the transition between media and adventitia. The distance between those two acoustic interfaces is the definition for IMT [3,4]. The average signal coming from the probe is converted in measurements by the software of the echographic system: the operator should only read the numbers on the screen in order to detect the IMT value in real time. Such an approach reduced the bias coming from manual measures just because the radio-frequency signal was converted directly into IMT value with limited interferences coming from operator skills. In particular, the ^RF^QIMT calculation was evaluated on temporal averaging of the RF signals received over cardiac cycle, obtaining a median value of the IMT over a few subsequent cardiac cycles in order to improve the accuracy of the technique. Figure 1 shows a sample case of ^RF^QIMT evaluation.

Experienced sonographer obtained images of the far wall of both CCA and carotid bulbs according to the Mannheim common carotid IMT consensus [4]. Thickness greater than 0.9 mm was regarded as increased CCA IMT. We re-evaluated 30 patients re-assessing IMT by RF even by a second ultrasonographer, in order to assess inter- and intra-observer variability which were both 0.96 and 0.98 respectively according to the intraclass correlation coefficient (classified as good if it is over 0.80 [24]).

### Echocardiography

All patients underwent echocardiographic examination by a single well-experienced ultrasonographer in order to reduce bias. Two dimensional images were obtained using standard views in the left lateral decubitus position. Images were acquired at passive end-expiration to minimize global cardiac movement from standard parasternal long axis and apical planes. Left ventricle (LV) dimensions were obtained in the standard views. LV end systolic and end diastolic volumes were calculated by using the modified Simpson's method, and ejection fraction was calculated from the LV end systole and end diastolic volumes. The left ventricular mass index (LVMI) (g/m^2^) was calculated using the Devereux's formula [25] by the following equation: Left Ventricular Mass (LVM) = 0.80 [1.04 × (interventricular septal thickness + posterior wall thickness + end-diastolic diameter) 3 - (end-diastolic diameter)3] + 0.6. The LVMI was calculated as LVM divided by the body surface area (BSA). LVMI is calculated according to end-diastolic measurements of LV posterior and septal wall thickness and internal dimension, standardizing all to body surface area.

We re-evaluated 30 patients re-assessing LVMI even by a second ultrasonographer, in order to assess inter- and intra-observer variability which were both 0.89 and 0.93 respectively according to the intraclass correlation coefficient (classified as good if it is over 0.80 [24]).

### Coronary angiography and assessment of coronary artery disease severity

Coronary angiography was performed via the femoral or radial artery using standard technique [17]. The choice of artery come from the decision of the operator and the conditions of the patient (for example, severe peripheral artery disease).

### Statistical Analysis

The data are given as mean values ± standard deviation (SD), and categorical variables as frequencies and percentage. Between-group comparisons were made using analysis of variance (ANOVA). The correlation of the studied variables to IMT was assessed using Pearson's coefficient, and multiple regression analysis was then applied to evaluate independent associations of the variables with ^RF^QIMT values. The coefficient of determination (R^2^) was used to measure the proportion of variability of the dependent variable that is attributable to the independent variables. Frequencies were compared using the chi-squared. A P value of < 0.05 was considered statistically significant. The statistical analyses were made using Statistica 6.1 software (StatSoft Inc., Tulsa, OK, USA).

## Results

According to coronary angiograms, we divided patients in relation to their severity of coronary disease and extent of the vessels damages (i.e., number of major epicardial vessels with ≥ 70% stenosis). In this way we recognize three groups of patients:

- monovasal one: only one vessel involved (79 patients, 69%)

- bi-vasal one: two coronaries characterized by ≥ 70% stenosis (24 patients, 21%)

- tri-vasal group: three major epicardial coronary arteries damaged (12 patients, 10%).

Before coronary angiography, all patients underwent two-dimensional echo-color-Doppler of the carotid arteries, adopting a high definition vascular echograph: Esaote MyLab 50 X-Vision, in order to detect with radio-frequency the IMT of carotid artery. ^RF^QIMT values obtained led us to divide our population in three tertiles: a) IMT ≥ 0.9 mm (37 patients, 32.2%); b) IMT > 0.91 mm and < or 1.19 mm (38 patients, 33%); c) IMT ≥ 1.2 mm. (40 patients, 34.8%).

All patients revealed plaques at the levels of internal carotid artery. Plaque was defined as a focal structure encroaching into the arterial lumen of at least 0.5 mm or 50% of the surrounding IMT value, or demonstrated a thickness greater than 1.5 mm as measured from the media-adventitia interface to the intima-lumen interface [3]. The plaques determined several degrees of stenosis (data not showed). In fact, mean percentage values between right and left internal carotid arteries stenosis are the following: 22 patients showed a stenosis < 40%; 59 a stenosis degree within 41-60%; 21 a stenosis degree within 61-80%; 13 a stenosis degree > 80%.

We related these latest values to demographic and clinical characteristics of the study population, as summarized in Table 2. We noticed that there were no significant differences in age, male gender, hypertension, diabetes, smoking, familiarity. Only dyslipidaemia seemed to be related to IMT value inducing a significant increasing of carotid wall thickness (p = 0.004) in our population.

**Figure 2:**
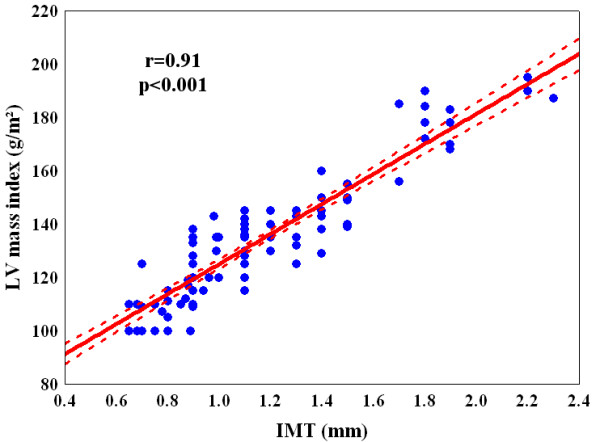
**Linear correlation between carotid artery intima media thickness (IMT) and left ventricular mass index (LVMI)**.

**Figure 3:**
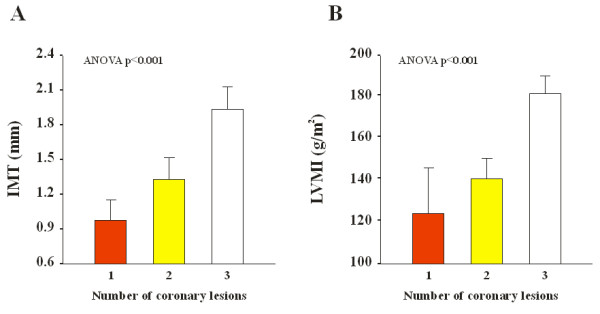
**Mean values of carotid artery intima media thickness (IMT; Panel A) and and left ventricular mass index (LVMI; Panel B) according to the number of coronary lesions**.

Our results show a highly statistically significant correlation between the extent of the number of coronary artery with a significant obstruction and the increase of ^RF^QIMT (P < 0.001) (Table 2). Also the correlation between the increase of LVMI and the growing of IMT was statistically significant (r = 0.91; P < 0.001) (Figure 2). It is therefore evident that a statistically significant association between the increase of IMT (Figure 3 panel A) and the rise of LVMI (Figure 3 panel B) is a predictor of important coronary lesions (P < 0.001).

In a multivariate regression model (R^2 ^= 0.89, see table 3), ^RF^QIMT remained significantly associated only with the dyslipidaemia (regression coefficient ± standard error [SE]: 0.049 ± 0.025; p = 0.05), LVMI (regression coefficient ± SE: 0.011 ± 0.001; P < 0.0001) and number of damaged coronaries (regression coefficient ± SE: 0.173 ± 0.034; P < 0.0001). Even considering a multivariate regression model (R^2 ^= 0.88, see table 3) only considering the upper three elements as independent determinants of ^RF^QIMT, the association remained statistically significant (dyslipidaemia regression coefficient ± SE: 0.057 ± 0.023, p = 0.017; LVMI: 0.01 ± 0.001, P < 0.0001; number of damaged coronaries: 0.0174 ± 0.028, P < 0.0001).

**Table 3:** Multivariate regression analysis of common carotid artery intima-media thickness evaluated by radio-frequencies [model 1: R^2 ^= 0.89; model 2: R: 0.88]

Model 1	Coefficient of regression ± SE	p
Age	0.001 ± 0.001	0.202
Male gender	-0.007 ± 0.028	0.813
Diabetes	0.012 ± 0.028	0.655
**Dyslipidaemia**	**0.049 ± 0.025**	**0.050**
Hypertension	0.039 ± 0.03	0.206
Smoking habits	0.006 ± 0.033	0.844
End-diastolic diameter	-0.006 ± 0.005	0.190
End-systolic diameter	0.003 ± 0.003	0.249
Interventricular septum	-0.003 ± 0.015	0.848
Posterior wall	-0.02 ± 0.017	0.244
**LV mass index**	**0.011 ± 0.001**	**< 0.0001**
LV ejection fraction	0.001 ± 0.002	0.432
**Number of stenosed coronary arteries**	**0.173 ± 0.034**	**< 0.0001**

**Model 2**		

**Dyslipidaemia**	**0.057 ± 0.023**	**0.017**
**Number of stenosed coronary arteries**	**0.01 ± 0.001**	**0.000**
**Number of stenosed coronary arteries**	**0.174 ± 0.028**	**< 0.0001**

S.E.: standard error; LV: left ventricle

## Discussion

CCA-IMT is a well-established surrogate marker of coronary atherosclerosis [26,27] and is associated with cardiovascular events [28]. It is efficient, relatively unexpensive and highly reproducible and does not expose patients to contrast dye or radiation. The adoption of RF improved the assessment of carotid evaluations as literature data [9-11] previously outlined. As Bianchini et al. [11] rightly pointed out in their work, RF assessment of CCA-IMT is similar to ultrasound one. So, although no reference values had been developed, the reproducibility of the two techniques allow us to adopted the same reference values. A recent and interesting review by O'Leary and Bots [29] pointed out the great advantages coming from automated edge detection of CCA-IMT, but it outlined the need of a "clear" ultrasound interface in order to achieve a perfect CCA-IMT value by an automated edge detection program. Naturally, when we assessed ^RF^QIMT we adopted the most clear images of common carotid arteries, using only the best images to perfume RF evaluation of IMT.

Previous studies demonstrated the relationship between CCA-IMT and the extent and severity of coronary stenosis [8], although none consider the adoption of RF to assess IMT, using the classical ultrasound to chase their aims. Carotid IMT has been shown to correlate with left ventricular mass in patients with hypertension [14,30] and we tried to point out such a relationship using RF in CCA-IMT evaluation, rather than simple ultrasound scans. LVH, defined as an increase in left ventricular mass, is another independent risk factor for cardiovascular morbidity and mortality in hypertensive patients, due to increased afterload imposed by hypertension, or to an asymptomatic coronary atherosclerosis (related to CCA-IMT increase [31]) able to create a transient experimental coronary occlusion which stimulates myocardial growth [32], and, consequentially, ventricular mass in patients with coronary stenosis [33,34]. The hypertensive status of our patients could reduce the data coming from LVMI, but we preferred to adopt a parameter less influenced by an acute onset of a coronary syndrome (as for example ejection fraction) and already standardized by international literature. It could be postulated that carotid IMT could be expression of a vascular hypertrophy in the absence of plaques. But all patients in our population sample showed carotid plaques causing different stenosis degree, as outlined in the results section. The multivariate regression analysis even showed no influence of hypertension status of our patients on our results. Nevertheless, Manios et al. [35] evaluate LVM and IMT in pre-hypertensive patients. It is really interesting to consider the association between such parameter in patients not already suffering from full hypertensive status. Nevertheless, these authors did not consider RF to evaluate CCA-IMT, which is already measured by ultrasound. Besides, although this study suggests a relationship between LVM and IMT before a full hypertensive status, in our study we consider LVMI because it is known to be related to CAD and it is more suitable to be considered as a marker of early atherosclerosis than others echocardiographic parameter.

In this study we have used ^RF^QIMT method in order to detect the CCA-IMT. This is an important technique able to measure carotid intima-media thickness and to reduce the bias coming from the manual measurements of this cardiovascular risk marker [9-11]. In fact, we have already pointed out the high accuracy and reproducibility of radiofrequencies in early detection of vessels walls abnormalities [9]. Literature data [9,22,23] underlined the superiority of ^RF^QIMT against the standard evaluation of CCA-IMT. The method, thanking technology and software evolution, is able to overcome the problems coming from inter- and intra-operator variabilities, although it is not able to wholly delete the entire bias. Schreuder et al. [10] underlined the reproducibility of both techniques (i.e., B-mode and RF), showing a good association between themselves. The methods has been highly reproducible in our case (inter- and intra-observer variability were both 0.96 and 0.98). The lacking of a simple ultrasound evaluation in order to compare the two methods could be considered as a limitation of the study, but we tried to compare the data coming from international literature, with those coming from our research which is the first comparing CCA-RFQIMT with LVMI and number of coronary arteries involved in high degree stenosis.

This points out the high accuracy of ^RF^QIMT in evaluating cardiovascular risk profile of patients at high risk for heart attack cause of their own coronaries situations. Therefore, it can be postulated that ^RF^QIMT provides diagnostic clue for ischemic etiology in severe LVH patients. We know from the few literature data [14] discovered, that there is a certain relationship between CCA-IMT and LVH in patients affected by CAD. Our work, at the best of our knowledge, is the only one that tries to underline the relationship between the ^RF^QIMT and LVMI (i.e., left ventricle mass adjusted for body surface) in patients with important stenosis in their own coronary vessels (our patients had to have a stenosis ≥ 70% in at least one major epicardial coronary artery). In particular, as Figure 1 points out, the association is statistical significant, with a p < 0.001, and positive, i.e. with increasing of carotid wall thickness, ventricular mass increases and this leads to an augmentation of risk of coronary artery diseases. The association is very high (r = 0.91) and received more importance by the ANOVA analysis of each parameter considered with the extent of coronaries damages. In fact, as Figures 3A and 3B underlined, both ^RF^QIMT and LVMI are significant associated with number of coronary artery obstructed (P < 0.001), the three coronaries involved much more dangerous than the one or two coronaries occluded. These data mean that, with increasing of LVMI and carotid IMT values, there is a clear augmentation of the risk to detect patients with several major epicardial coronary vessels showing stenosis > 70%.

## Conclusions

At the best of our knowledge, this is the first work that points out the importance of both ^RF^QIMT and LVMI related to coronary artery disease, in a population of patients suffering from severe (≥ 70%) coronary stenosis of at least one major epicardial coronary vessel.

^RF^QIMT detection once more outlined the relationship with cardiovascular risk profile of patients: its increase is related to number of stenosed coronary vessels and LVMI, a well-established marker of cardiovascular risk. The considering of a population with an advanced atherosclerotic process at coronary level could be considered as a limitation, but, evaluating the ^RF^QIMT before coronary angiography, we would like to point out the accuracy of this latest methods in the general assessment of number of coronary vessels involved in coronary artery disease.

## List of abbreviations

BSA: body surface area; CAD: coronary artery disease; CCA-IMT: common carotid artery intima media thickness; DBP: diastolic blood pressure; IMT: intima media thickness; LV: left ventricle; LVH: left ventricular hypertrophy; LVM: left ventricular mass; LVMI: left ventricular mass index; NSTEMI: non-ST segment elevation myocardial infarction; RF: radio-frequency; ^RF^QIMT: Radio Frequency-Quality Intima Media Thickness; SBP: systolic blood pressure; SD: standard deviation; SE: standard error; STEMI: ST segment elevation myocardial infarction

## Competing interests

The authors declare that they have no competing interests.

## Authors' contributions

MMC conceived and designed the study, analysed and interpreted the data, drafted the article and critically reviewed its intellectual content, and finally approved the version to be submitted for publication; FC measured and calculated intima-media thickness via radio-frequencies methods, reviewed the article's intellectual content, and finally approved the version to be submitted for publication; LA measured and calculated echocardiographical data, reviewed the article's intellectual content, and finally approved the version to be submitted for publication; PS, AZ, MG, RC analysed the data, reviewed the article's intellectual content, and finally approved the version to be submitted for publication; SM, FF contributed towards designing the study, interpreting the cardiological data, critically reviewing the article's intellectual content, and finally approving the version to be submitted for publication.
